# Evolution of anatomical characters in *Acianthera* section *Pleurobotryae* (Orchidaceae: Pleurothallidinae)

**DOI:** 10.1371/journal.pone.0212677

**Published:** 2019-03-13

**Authors:** Audia Brito Rodrigues de Almeida, Eric de Camargo Smidt, Erika Amano

**Affiliations:** Programa de Pós-Graduação em Botânica, Setor de Ciências Biológicas, Universidade Federal do Paraná, Curitiba, PR, Brazil; Fred Hutchinson Cancer Research Center, UNITED STATES

## Abstract

*Acianthera* section *Pleurobotryae* is one of ten sections of the genus *Acianthera* and include four species endemic to the Atlantic Forest. The objective of this study was to describe comparatively the anatomy of vegetative organs and floral micromorphology of all species of *Acianthera* section *Pleurobotryae* in order to identify diagnostic characters between them and synapomorphies for the section in relation of other sections of the genus. We analyzed roots, ramicauls, leaves and flowers of 15 species, covering eight of the nine sections of *Acianthera*, using light microscopy and scanning electron microscopy. *Acianthera* section *Pleurobotryae* is a monophyletic group and the cladistic analyses of anatomical and flower micromorphology data, combined with molecular data, support internal relationship hypotheses among the representatives of this section. The synapomorphies identified for *A*. sect. *Pleurobotryae* are based on leaf anatomy: unifacial leaves, round or elliptical in cross-section, round leaves with vascular bundles organized in concentric circles, and mesophyll with 28 to 30 cell layers. Within the section, the clade (*A*. *crepiniana* + *A*. *mantiquyrana*) presented more differences in vegetative organ morphology and higher support values in combined analyses when compared to the second clade, (*A*. *atropurpurea* + *A*. *hatschbachii*). For each of these clades an exclusive set of homoplasies of vegetative and floral organs were also identified. The results support the argument that vegetative organs are more evolutionarily stable in comparison to reproductive organs and thus helpful for inference of internal phylogenetic relationships in *Acianthera*, while flowers are highly variable, perhaps due to the diversity of pollinator attraction mechanisms. The analyses indicate that the elliptical leaves observed in *A*. *crepiniana* have originated from round leaves observed in the other species of this section, suggesting an adaptation to increase the area of exposure of the leaf and better the efficiency of capture of sunlight in shaded environments such as the Atlantic Forest. The presence of papillose regions in both vegetative and floral organs indicated that micromorphological characters are also useful for the delimitation of species and sections within the genus.

## Introduction

The family Orchidaceae represents approximately 7% of all angiosperms, and half of the Monocotyledons [1], with approximately 25,000 species and approximately 800 genera [2]. Orchids are found worldwide, but the greatest diversity, including nearly all of the epiphytic species, occurs in the tropics, especially in tropical mountains [3]. These species often establish complex interactions with other organisms for their growth, development and reproductive success. Therefore, they are highly susceptible to environmental change and play an important ecological role in ecosystems [4]. Some orchids are used for commercial purposes as ornamental plants, food and as medicinal plants. [5].

*Acianthera* Scheidw. is an orchid genus in the neotropical subtribe Pleurothallidinae Lindl. Ex G. Don (Epidendreae: Epidendroideae: Orchidaceae), which presents its highest species diversity in the rainforests of Costa Rica, Panamá, Colombia, Equador, Venezuela, Peru and Brazil [6–8]. *Acianthera* currently includes 291 species of terrestrial, rupicolous and especially epiphytic plants with long-repent or repent growth and variable size found throughout South America [8, 9]. The largest number of species is found in Brazil [10], many of them endemic [11].

The placement of some Pleurothallidinae genera, including *Acianthera*, was revised by Luer, who proposed an intrageneric classification system for *Pleurothallis* R. Br. based entirely on morphological characters [8]. The circumscription of *Pleurothallis* was then expanded to include the species of *Acianthera* [8]. However, later phylogenetic analyses using nuclear and plastid DNA indicated that *Pleurothallis lato sensu* was not a monophyletic group [12], although *Pleurothallis* subgenus *Acianthera* did form a clade. Therefore, *Acianthera* was re-established, making *Pleurothallis stricto sensu* also monophyletic [12].

After a taxonomic revision and phylogeny based on molecular analyses of nuclear ITS sequences, *Acianthera* was divided in ten sections, including the new *Acianthera* section *Pleurobotryae* (Barb. Rodr.) Chiron & van den Berg [13]. This section was proposed with seven species that were previously under the genus *Pleurobotryum* Barb. Rodr. [13]. Currently, *Acianthera* section *Pleurobotryae* is recognized with four species of epiphytic species from higher regions of the Atlantic Forest, found between 100 and 2,000 m, characterized by cylindrical or laterally compressed leaves: *Acianthera atropurpurea* (Barb. Rodr.) Chiron & van den Berg, *Acianthera crepiniana* (Cogn.) Chiron & van den Berg, *Acianthera hatschbachii* (Schltr.) Chiron & van den Berg and *Acianthera mantiquyrana* (Barb Rodr.) V.T. Rodrigues & F. Barros [14]. Recent phylogenetic analyses, based on nuclear ITS and chloroplast matK sequences, recovered *A*. section *Pleurobotryae* as a strongly supported monophyletic group and the group diagnosis requires the occurrence of cylindrical or laterally compressed leaves along with a filiform caulome [15].

**Fig 1:**
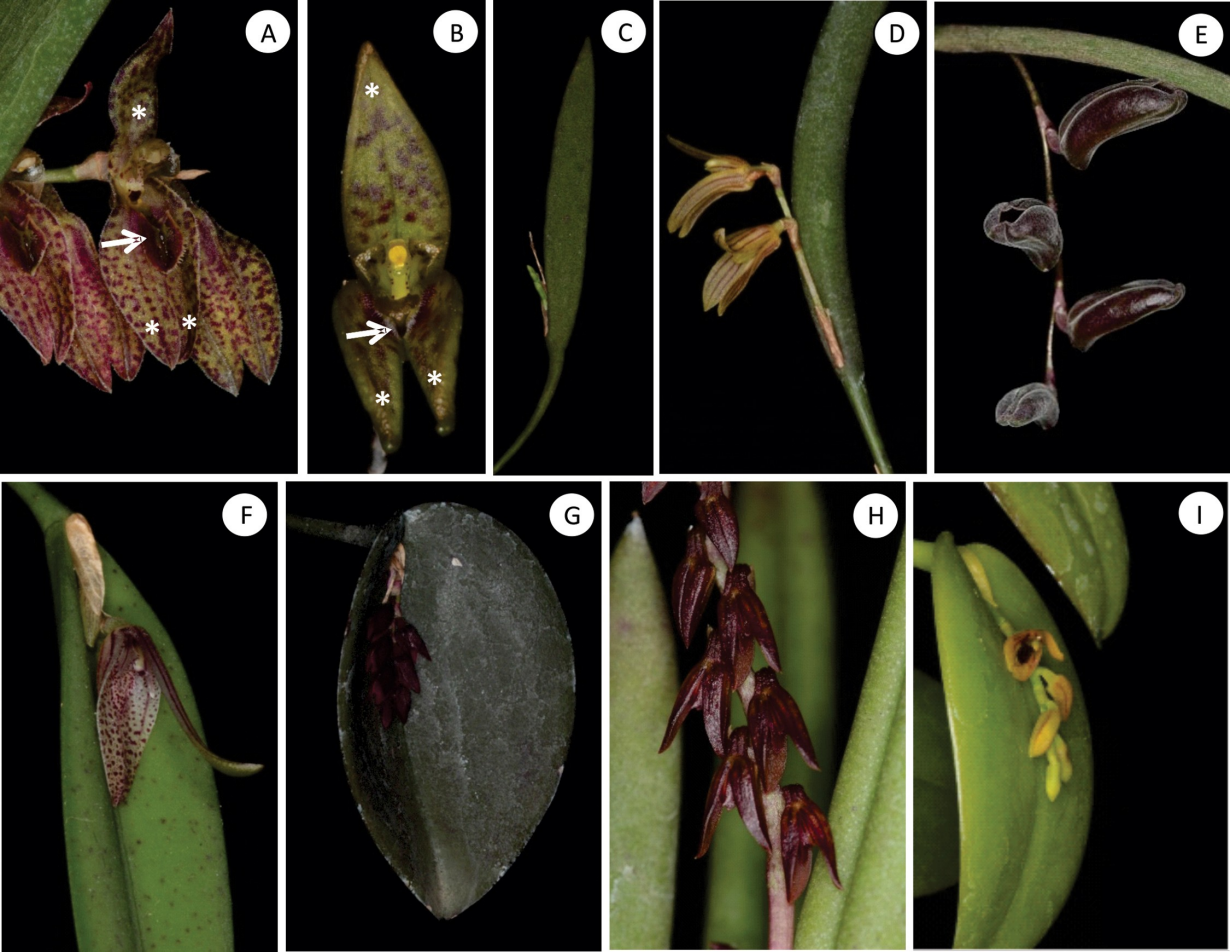
Morphological aspects of sampled *Acianthera*. *Acianthera* section *Pleurobotryae* (A-E), *Acianthera* section *Acianthera* (F), *Acianthera* section *Sicariae* (G), *Acianthera* section *Tricarinatae* (H), *Acianthera* section *Sulcatae* (I). (A) *Acianthera crepiniana* flower showing sepals (*) and labellum (arrow). (B) *Acianthera hatschbachii* flower showing sepals (*) and labellum (arrow). (C) *Acianthera crepiniana* vegetative organs, ramicaul and leaf. (D) *Acianthera mantiquyrana*. (E) *Acianthera atropurpurea*. (F) *Acianthera gracilisepala*. (G) *Acianthera prolifera*. (H) *Acianthera teres*. (I) *Acianthera luteola*.

Anatomical studies [16–18] in conjunction with floral micromorphology [19–22] have recently proved to be important tools that allowed a better understanding of phylogenetic relationships within Orchidaceae. The anatomy of vegetative organs of some *Acianthera* and other Pleurothallidinae has been described [6, 23–28]; however, studies placing anatomical characters in a phylogenetic framework are rare [6, 29]. The morphology of both vegetative and floral organs is highly variable in *Acianthera* [8, 13, 30] (Fig 1). Most sections of the genus *Acianthera* were recently delimited based on molecular studies [13]. However, species of different sections and habits may have similar morphological characters, whereas species circumscribed in the same section may present different morphological characters. This great variation makes it difficult to position certain species within the sections.

The objectives of this study were: 1) to describe comparatively the anatomy of vegetative organs and floral micromorphology of all species of *Acianthera* section *Pleurobotryae* in order to identify diagnostic characters for taxonomy at both section and species levels; 2) to identify synapomorphies for the *A*. sect. *Pleurobotryae* relative to the other sections of *Acianthera*.

## Materials and methods

### Taxon sampling

A total of 15 species of *Acianthera* was studied, covering eight of the nine sections of this genus (Table 1). The species *Pleurothallopsis nemorosa* Porto & Brade was included as outgroup, based on recent phylogenetic studies of the section [15].

**Table 1:** *Acianthera* specimens used for analysis of the vegetative organs anatomy and floral micromorphology.

Taxon	Habit	Section	Voucher	GenBank access number (nrITS)/(cpDNA matK)
*Pleurothallopsis nemorosa* (Barb. Rodr.) Porto & Brade	epiphytic	Outgroup	Toscano de Brito, A.L.V. 3414 UPCB	KT599880 / KT709650
*Acianthera luteola* (Lindl.) Pridgeon & M. W. Chase	epiphytic	*Sulcatae*	Almeida, A.B.R. 011, 026, 025 HUCP	KX495754 / KT709640
*A*. *octophrys* (Rchb. f.) Pridgeon& M. W. Chase	epiphytic	*Tomentosae*	Toscano de Brito, A. L. V. 3410 UPCB	KT599879 / KT709643
*A*. *fenestrata* (Barb. Rodr.) Pridgeon& M. W. Chase	epiphytic	*Cryptophoranthae*	Almeida, A.B.R. 036 HUCP	AF262857 / KT709635
			Klein, J. 067 UPCB	
*A*. *hystrix* (Kraenzl.) F. Barros	epiphytic	*Crinitae*	Toscano de Brito, A. L. V. 2893 UPCB	KT599877 / KT709639
*A*. *atropurpurea* (Barb .Rodr.) Chiron & van den Berg	epiphytic	*Pleurobotryae*	Almeida, A.B.R. 020 HUCP	KT599874 / KT709633
*A*. *hatschbachii* (Barb. Rodr.) Chiron & van den Berg	epiphytic	*Pleurobotryae*	Imig, D.C. 445 UPCB	KT599876 / KT709638
			Kersten, R. A. HUCP 18411	
			Almeida, A.B.R. 03, 021 HUCP	
*A*. *crepiniana* (Cogn.) Chiron & van den Berg	epiphytic	*Pleurobotryae*	Almeida, A.B.R. 05 HUCP	KT599875 / KT709634
			Imig, D.C. 444 UPCB	
*A*. *mantiquyrana* (Barb. Rodr.) V. T. Rodrigues & F. Barros	epiphytic	*Pleurobotryae*	Almeida, A.B.R. 02 HUCP	KT599878 / KT709641
*A*. *saurocephala* (Lodd.) Pridgeon& M. W. Chase	epiphytic	*Acianthera*	Almeida, A.B.R. 07, 027 HUCP	JQ306356 / KT709648
*A*. *gracilisepala* (Brade) Luer	epiphytic	*Acianthera*	Almeida, A.B.R. 01 HUCP	JQ306404 / KT709636
*A*. *pubescens* (Lindl.) Pridgeon & M. W. Chase	epiphytic	*Acianthera*	Almeida, A.B.R. 04, 028 HUCP	JQ306366 / KT709645
			Oliveira, L.R. L 022, 029 UPCB	
*A*. *saundersiana* (Rchb.f.) Pridgeon& M. W. Chase	epiphytic	*Sicarie*	Almeida, A.B.R. 034 HUCP	JQ306452 / KT709647
*A*. *prolifer*a (Herb. ex Lindl.) Pridgeon & M. W. Chase	epiphytic	*Sicarie*	Almeida, A.B.R. 014 HUCP	AF275688 / KT709644
			Koene, F.M. 001 UPCB	
*A*. *ochreata* (Lindl.) Pridgeon& M. W. Chase	rupicolous	*Tricarinatae*	Almeida, A.B.R. 09 HUCP	JQ306427 / KT709642
			Koene, F.M. 003 UPCB	
*A*. *teres* (Lindl.) Borba	rupicolous	*Tricarinatae*	Almeida, A.B.R. 015 HUCP	AF366937 / KT709649

### Light microscopy and scanning electron microscopy

Samples of root, middle third of ramicauls and the leaf blade, fully developed, were fixed in FAA (37% formaldehyde, glacial acetic acid, 50% ethanol; 1:1:18 v:v:v) [31]. The material was sectioned by hand using a razor blade, stained with alcian blue and basic fuchsin [32] and mounted in glycerol gelatin [33]. Root samples from 2 to 3 cm from the root apex were processed in Leica Historesin according to the manufacturer's instructions and stained with toluidine blue [34]. Histochemical tests were performed for identification of starch (Lugol solution, potassium iodide) [31], lignin (acidic phloroglucinol) [35] and lipids (Sudan III) [36]. The dissociation of the leaf epidermis was perfomed using a 1:1 solution of glacial acetic acid and hydrogen peroxide at 60°C for 12 hours [37] and stained with alcoholic safranin at 50% [38] for observation of the stomatal type, stomatal distribution and epidermis cells. The autofluorescence of wall thickenings of tracheoidal idioblasts was recorded by fluorescence microscopy [39] using RFP or Texas red filters and by confocal microscopy using a Nikon A1RsiMP microscope. Images were visualized using the software NisElements 4.20.

Leaves fixed in FAA and flowers at second day of anthesis fixed in 2.5% (v/v) glutaraldehyde/4% (v/v) formaldehyde in phosphate buffer (pH 7.4; 0,1M) for 2 hours were dehydrated in ethyl alcohol series, critical point dried and coated with gold [40]. Electron micrographs were produced using a TESCAN VEGA3 LMU scanning electron microscope.

### Terminology

Morphological descriptions follow Stern [5], Luer [8] and Chiron and van den Berg [13]. The description of the epicuticular wax deposition patterns follows the terminology proposed by Barthlott et al. [41]; cell wall thickness classification follows that proposed by the IAWA Committee [42]; shape of the epidermal cells in frontal view follows Koch, Bhushan & Barthlott [43]. The classification of epidermal appendages was generally adapted from Davies & Turner [44] and Davies & Stpiczyn´ska [45]; the classification of unique epidermal appendages was adapted from that proposed by Stearn [46] for spores.

### Cladistic analyses

The character matrix was assembled using the program Mesquite 3.1 [47]. Morphological character construction and coding followed Sereno [48]. Quantitative characters were coded based on the model proposed by Thiele [49]. All characters were equally weighted and with unordered states [50]. Molecular data for nuclear (nrITS) and plastidial (cpDNA matK) DNA were obtained from Rodrigues et al. [15] (Table 1).

Maximum parsimony analyses were conducted using PAUP 4.0b10a [51] with 1,000 replicates of random taxon addition sequences, keeping 10 trees per replicate, using TBR branch swapping, followed by a second search to explore more topologies based on the first search, limited to 10,000 trees. Support was estimated from 1,000 bootstrap replicates (BP) [52] with simple addition sequence using TBR and 10 trees per replicate.

Phylogenetic relationships were also evaluated through Bayesian inference using Mr Bayes 3.1 [53]. Evolution models used for molecular data follow Rodrigues et al. [15]: K80+G for ITS1, K80 for 5.8S and HKY+G ITS2 (nrITS) and GTR+G for matK. The model used for the morphological data partition was mk1 [54]. The Markov chain Monte Carlo (MCMC) was started from random trees and run for 10 million generations, sampling every 1,000. The first 25% generations were discarded as burn-in after visual inspection of the logarithmic probability of the trees as indicated by the Stdev(s) and PSRF [55]. The remaining trees were used to produce a majority consensus tree with the posterior probability values (PP), visualized in Tree View [56]. The strict consensus tree resulting from the total evidence maximum parsimony analysis was visualized using Winclada [57] with accelerated transformation (ACCTRAN) reconstruction to facilitate identification of apomorphies and homoplasies for each resulting clade.

## Results

### Root

**Fig 2:**
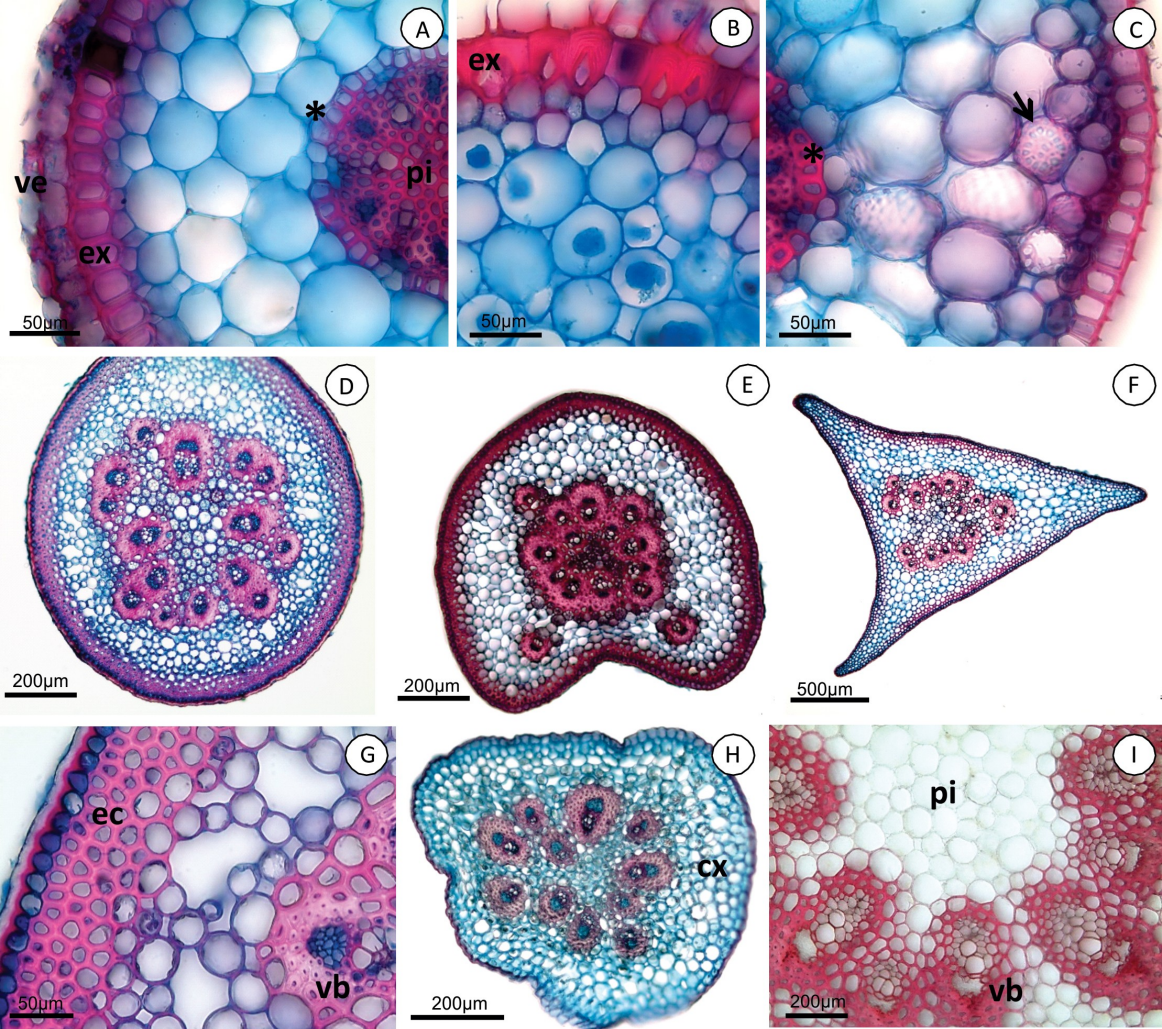
***Acianthera* root (A-C) and ramicaul (D-I) cross-sections.** (A) *Acianthera luteola*. Bi-layered velamen (ve), exodermis with O-shaped wall thickening (ex) and endodermis with U-shaped thickening (*), pith cells (pi) with thickened lignified walls. (B) *A*. *crepiniana*, exodermis with inverted-U shaped thickening (ex). (C) *A*. *fenestrata*, cortex with reticulate idioblasts (arrow) and endodermis with O-shaped thickening (*). (D) *Acianthera crepiniana*, round ramicaul. (E) *Acianthera saundersiana*, round-sulcate ramicaul. (F) *Acianthera luteola*, triangular ramicaul. (G) *Acianthera crepiniana*, external cortex (ec) with sclerified cells. (H) *Acianthera hystrix*, ramicaul with parenchymatous cortex (cx). (I) *Acianthera pubescens*, ramicaul pith with unlignified cells (pi) after acidified phloroglucinol reaction. Vascular bundle (vb).

The roots are fasciculate, thin, cylindrical and velamentous, and have approximately 1–2 mm in diameter at their maximum thickness. All species analyzed presented a bi-layered velamen (Fig 2A). *Acianthera* section *Pleurobotryae* species present exodermis cells lignified with an inverted-U shaped wall thickening (Fig 2B) and a parenchymatous cortex with eight to nine cell layers. In the other sections, the species present exodermis cells with an inverted-U or O-shaped thickening and parenchymatous cortex with seven to nine cell layers.

Among the species in *A*. section *Pleurobotryae*, only *A*. *hatschbachii* did not present raphides in the cortex. The endodermis in *A*. section *Pleurobotryae*, as well as in most other species analyzed, is lignified and presents O-shaped wall thickening (Fig 2C), except in *A*. *mantiquyrana*, *A*. *luteola* (Lindl.) Pridgeon & Chase and *A*. *octophrys* Rchb. f., where the endodermis presents U-shaped thickening (Fig 2A). *Acianthera atropurpurea* and *A*. *mantiquyrana* endodermal cells are very thin-walled, while *A*. *crepiniana* and *A*. *hatschbachii* present thin- to thick-walled cells. In the other species, the endodermal cells vary from very thin-walled to very thick-walled. Passage cells in the endodermis appear in groups of one, two or three (Fig 2A and 2C). The number of protoxylem poles is variable: up to seven in *A*. *mantiquyrana*, nine in *A*. *hatschbachii*, and more than nine in *A*. *atropurpurea* and *A*. *crepiniana*. Tracheoidal idioblasts with reticulate lignified wall thickening were observed in the cortex of *A*. *hatschbachii* (*A*. section *Pleurobotryae*) and in the species of other sections of *Acianthera* (Fig 2C).

### Ramicaul

The ramicauls are slender, vary from very short to elongated, with one to several nodes and internodes and from terete to winged. The ramicaul in *A*. section *Pleurobotryae* is round in cross-section (Fig 2D), while in other species it can be round, round-sulcate (Fig 2E) or triangular (Fig 2F).

In cross-section the epidermis is uniseriate with dome-shaped cells (Fig 2G), except in *A*. *hystrix*, where the cells are irregular. The cuticle is thick (Fig 2G). The cortex presents 7 to 10 cell layers in *A*. section *Pleurobotryae*; in other species this number varies from six to 16 cells layers. The external cortex in *A*. *atropurpurea*, *A*. *hatschbachii* and *A*. *mantiquyrana* is constituted by three layers of sclerified cells; *A*. *crepiniana* presents five layers (Fig 2G). There are two to four layers in other species, and none in *A*. *hystrix* (Kraenzl.) F. Barros (Fig 2H). Species in *Acianthera* section *Pleurobotryae* usually present aerenchyma and starch grains in the cortex (Fig 2G); however, aerenchyma was not observed in *A*. *mantiquyrana* and starch grains were not observed in *A*. *hatschbachii*. The vascular system is formed by collateral bundles organized in concentric circles (Fig 2D–2F) in all species. The number of vascular bundles is variable: in *A*. sect. *Pleurobotryae* vary from 13 to 25, and in the other species it can vary from 11 to 50. The interfascicular fundamental tissue consists of sclerified cells in all species analyzed except for *A*. *hystrix*. The pith cells present lignified walls, except in *A*. *hystrix* and *A*. *pubescens* (Lindl.) Pridgeon & Chase (Fig 2H and 2I).

### Leaf

Leaves of *Acianthera* species are thick and fleshy, and can be erect, prostate or pendent. Leaves vary greatly in shape, from elliptical to linear, laterally flattened to acicular (Fig 1C–1I). Species in *A*. section *Pleurobotryae* present unifacial leaves whereas in the species of other sections the leaves are bifacial.

**Fig 3:**
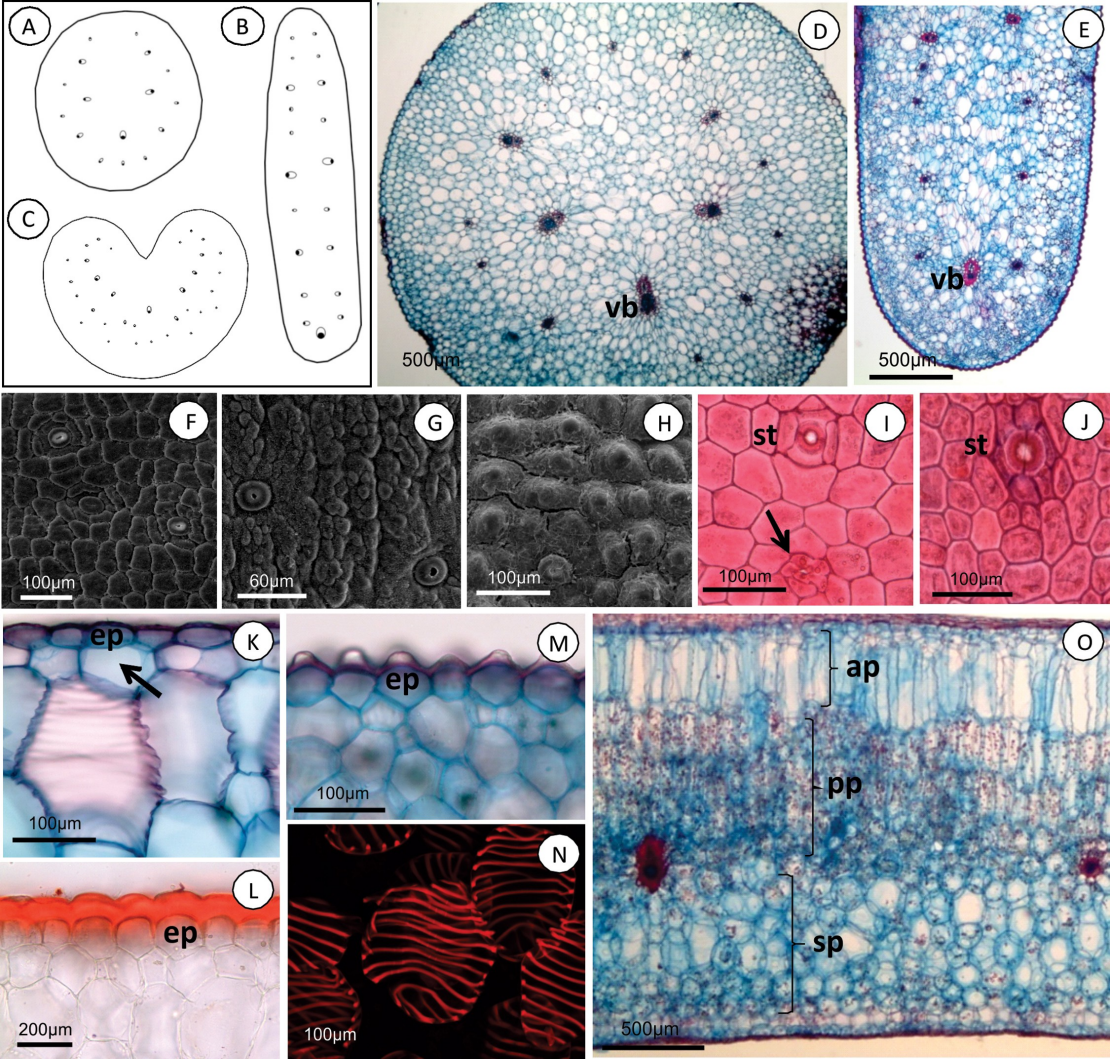
*Acianthera* leaf blades. **Schematic drawing of cross-sections (A-C), cross-sections under light microscopy (D and E), frontal view under SEM (F-H), and light microscopy (I-O).** (A) *A*. *atropurpurea*, line drawing highlighting organization of vascular bundles in mesophyll. (B) *A*. *crepiniana*, line drawing of elliptical leaf highlighting organization of vascular bundles in mesophyll. (C) *Acianthera teres*, round-sulcate leaf showing adaxially facing phloem. (D) *Acianthera mantiquyrana*, circular leaf, showing wider vascular bundles (vb) towards the medial region and outside-facing phloem. (E) *Acianthera crepiniana*, elliptical leaf with two parallel vascular bundles (vb) of larger diameter in basal area. (F) *Acianthera saurocephala*, smooth epidermis. (G) *Acianthera luteola*, verrucous epidermis. (H) *Acianthera crepiniana*, papillose epidermis. (I) *Acianthera atropurpurea*, epidermis with trichome insertion scars (arrow). (J) *Acianthera teres*, epidermis showing stomatal complex with six subsidiary cells. (K) *Acianthera saundersiana*, polygonal cells in epidermis (ep) and single-layered adaxial hypodermis (arrow). (L) *Acianthera teres*, dome-shaped epidermal cells (ep), and cuticle with Sudan III reaction. (M) *Acianthera crepiniana*, papillose epidermis (ep). (N) *Acianthera prolifera*, round tracheoidal idioblast in mesophyll. (O) *Pleurothallopsis nemorosa*, heterogeneous mesophyll with aquiferous parenchyma (ap), palisade parenchyma (pp) and spongy parenchyma (sp).

In cross-section, the leaves of *A*. *atropurpurea*, *A*. *hatschbachii* and *A*. *mantiquyrana* are round (Fig 3A and 3D), elliptical in *A*. *crepiniana* (Fig 3B and 3E) and round-sulcate (Fig 3C), flat or semi-flat in species of other sections. The leaf surface in frontal view is smooth in *A*. *atropurpurea* and *A*. *hatschbachii* (Fig 3F), verrucous in *A*. *saundersiana* and *A*. *luteola* (3G) or papillose in *A*. *crepiniana* and *A*. *mantiquyrana* (3H). In the other species the leaf surface is smooth or papillose. Trichome scars are present in the epidermis (Fig 3I, arrow), except in *A*. *luteola* and *Pleurothallopsis nemorosa*. All species presented hypostomatic leaves except *A*. *prolifera* (Herb. ex Lindl.) and *A*. *teres* (Lindl.) Borba, which presented amphistomatic leaves. The stomata consist of two guard cells surrounded by five subsidiary cells in *Acianthera* section *Pleurobotryae*, *A*. *fenestrata* (Barb. Rodr.) Pridgeon & Chase, *A*. *luteola* and *A*. *pubescens* (Fig 3I) and six subsidiary cells in other species (Fig 3J). In cross-section, the epidermal cells are polygonal (Fig 3K), dome-like (Fig 3L) or papillose (Fig 3M) and are covered by cuticle (Fig 3L and 3M). All species present a differentiated, single-layered hypodermis (Fig 3K, arrow), except for *A*. *crepiniana* (Fig 3M).

The mesophyll in the species of *A*. section *Pleurobotryae* presents 28 to 36 cell layers, with aquiferous parenchyma dispersed throughout (Fig 3D and 3E); in the species of other sections there are 13 to 35 cell layers. All species analyzed present a homogeneous chlorenchyma (Fig 3D and 3E), except for *P*. *nemorosa* (Fig 3O). The tracheoidal idioblasts are cylindrical or globose, with helicoidal lignification in the aquiferous parenchyma (Fig 3N), except in *P*. *nemorosa* and in the species of *A*. section *Pleurobotryae*, which do not have such structures. Species in *A*. section *Pleurobotryae* presented vascular bundles organized concentrically (Fig 3A and 3D) or in two rows parallel to the longest axis of the leaf (Fig 3B and 3E); in the species of other sections the vascular bundles are organized in one to three rows (Fig 3C).

### Sepals

**Fig 4:**
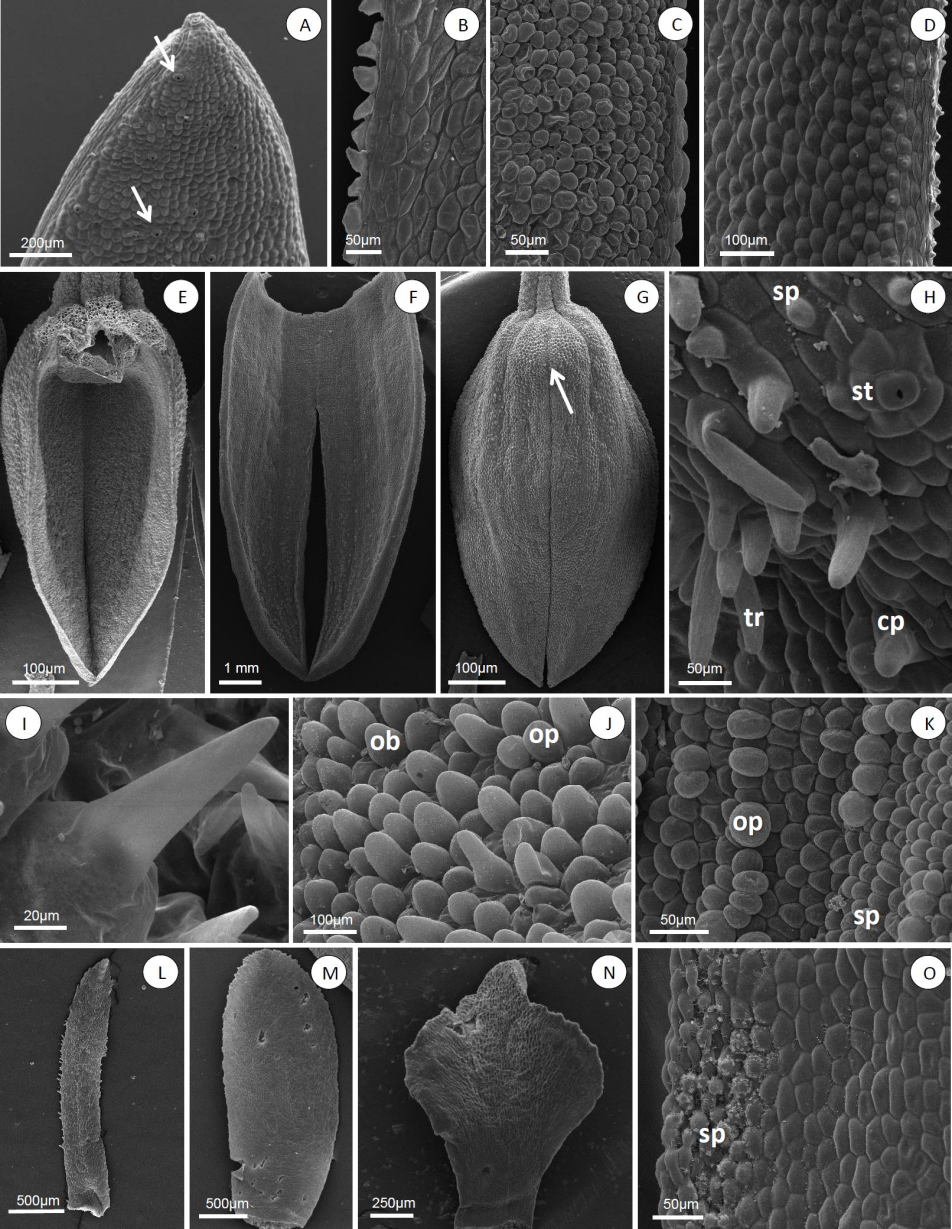
***Acianthera* and *Pleurothallopsis nemorosa*, floral micromorphology: dorsal sepals (A-D), lateral sepals (E-G) papillae and trichomes (H-K), petals (L-O).** (A) *Acianthera gracilisepala*, sepal apex with stomata (arrow). (B) *Acianthera crepiniana*, ciliate entire margin. (C) *Acianthera saundersiana*, papillose entire margin. (D) *Acianthera atropurpurea*, papillose delimited margin. (E) *Acianthera fenestrata*. (F) *Pleurothallopsis nemorosa*. (G) *Acianthera saundersiana*. (H) *Acianthera octophrys*, stomata (st), simple trichomes (tr), conical papillae (cp) and simple papillae (sp). (I) *Acianthera atropurpurea*, aciculate apex papillae. (J) *Acianthera fenestrata*, obovate (ob) and obpyriform papillae (op). (K) *Pleurothallopsis nemorosa*, simple (sp) and obpyriform papillae (op). (L) *Acianthera mantiquyrana*. (M) *Pleurothallopsis nemorosa*. (N) *Acianthera saurocephala*. (O) *Acianthera hatschbachii*, simple papillae (sp) on petals.

The sepals of all species analyzed are fleshy and often pubescent externally. Most species analyzed presented dorsal sepals free from the lateral sepals (Fig 1A, 1B and 1D), except *A*. *atropurpurea* (Fig 1E), *A*. *fenestrata*, *A*. *hystrix*, *A*. *prolifera* (Fig 1F), *A*. *ochreata* (Lindl.) Pridgeon & Chase and *A*. *teres* (Fig 1G), where the sepals are partially coalescent. The margin of dorsal sepals may be entire or delimited, glabrous, ciliated or papillose (Fig 4A–4D). Various levels of coalescence in lateral sepals were observed (Fig 4E–4G). The lateral sepals in *Acianthera luteola*, *A*. *octophrys*, *A*. *fenestrata*, *A*. *saundersiana* (Rchb.f.) Pridgeon & Chase, *A*. *prolifera*, *A*. *ochreata* and *A*. *teres* present mentum (Fig 4G, arrow). Stomata were observed in sepals of all species analyzed (Fig 4A, arrow); *A*. *atropurpurea*, *A*. *octophrys*, *A*. *fenestrata* and *A*. *hystrix* have hypostomatic sepals, *A*. *hatschbachii* has epistomatic sepals, and the other species have amphistomatic sepals.

In frontal view, the epidermal cells have polygonal and polygonal-elongate shape (Fig 4A–4D). In most species, the cells in the apical region of the sepals were differentiated, except in *A*. *atropurpurea*, *A*. *hatschbachii*, *A*. *hystrix*, *A*. *luteola*, *A*. *mantiquyrana* and *A*. *octophrys*. The epidermal surface of the sepals in *A*. section *Pleurobotryae* and *A*. *ochreata* is papillose (Fig 4A, 4C and 4D), glabrous in *A*. *luteola* and with both papillae and trichomes in other species (Fig 4H). The species of *A*. section *Pleurobotryae* may present simple, conical (Fig 4D) or aciculate (Fig 4I) papillae; other species may also present ovate (Fig 4J), obovate (Fig 4A and 4J) or obpyriform (Fig 4K) papillae.

### Petals

Petals are membranous and reduced compared to the sepals in all analyzed species. In *A*. section *Pleurobotryae*, petals are oblanceolate with entire margins, except in *A*. *mantiquyrana*, which presents serrate margins (Fig 4L). In other species, petals may be oblanceolate (Fig 4M) or deltoid (Fig 4N), with entire or serrate margins.

In frontal view, the epidermal cells are polygonal or elongate polygonal. The petals have epidermal cells with straight periclinal walls and papillae in *A*. *atropurpurea*, *A*. *hatschbachii*, *A*. *pubescens* e *A*. *prolifera*; the other species have epidermal cells with straight periclinal walls. In *A*. *atropurpurea*, *A*. *pubescens* and *A*. *prolifera* the papillose regions were observed in the apical region of the petals; in *A*. *ochreata* and *A*. *teres* (Fig 4O), in the apical and medium region, and in *A*. *hatschbachii*, in the basal region.

### Labellum

**Fig 5:**
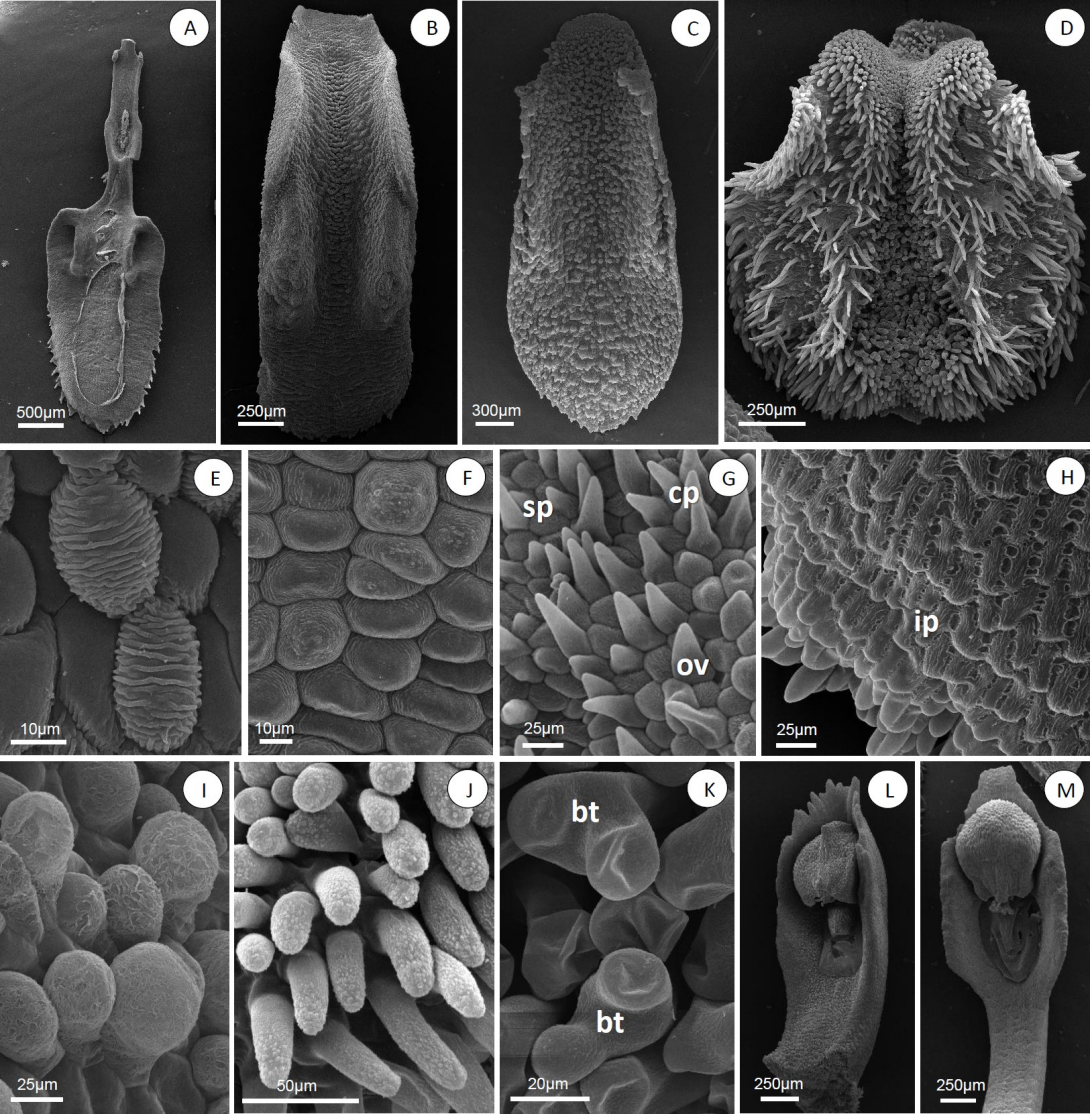
***Acianthera* and *Pleurothallopsis nemorosa*, labellum, general view (A-D), labellum micromorphology (E-K) and column, general view (L and M).** (A) *Acianthera hatschbachii*. (B) *Acianthera ochreata*. (C) *Acianthera prolifera*. (D) *Acianthera octophrys*. (E) *Acianthera octophrys*, elongate polygonal cells with transverse striations. (F) *Acianthera octophrys*, irregular cells with concentric striations. (G) *Pleurothallopsis nemorosa*, simple (sp), conical (cp) and ovate papillae (ov). (H) *Acianthera atropurpurea*, imbricate papillae (ip). (I) *Acianthera saundersiana*, obpyriform papillae (op). (J) *Acianthera octophrys*, simple trichomes. (K) *Acianthera octophrys* bifurcate trichomes (bt). (L) *Acianthera octophrys*. (M) *Acianthera ochreata*.

The labellum is thickened and ligulate in all species analyzed. Among the species of *A*. section *Pleurobotryae*, *A*. *atropurpurea*, *A*. *hatschbachii* and *A*. *mantiquyrana* presented an entire labellum (Fig 5A); *A*. *crepiniana* and the representatives of other sections presented a trilobed labellum (Fig 5B–5D). Lateral lobes are round (Fig 5B), except in *A*. *crepiniana*, *A*. *gracilisepala* (Brade) Luer, *A*. *pubescens*, *A*. *prolifera*, *A*. *saundersiana* and *A*. *teres*, where lateral lobes are apiculate (Fig 5C). The labellum presents a round apex and serrate margins in all species of *A*. section *Pleurobotryae* (Fig 5A); in other species the apex shape is round, obtuse, retuse, truncate or acute and the margins are entire, serrate or ciliate (Fig 5B–5D). The labellum surface is homogeneous in *A*. *pubescens*, *A*. *saundersiana* and in the species of *A*. section *Pleurobotryae* (Fig 5A), except for *A*. *atropurpurea*.

In frontal view, surface cells may be polygonal, polygonal-elongate (Fig 5E) or irregular (Fig 5F and 5G). The labellum in *A*. section *Pleurobotryae* presents simple and imbricate papillae (Fig 5H), while other species may present conical, ovate (Fig 5G), obpyriform (Fig 5I) papillae and various kinds of trichomes on the epidermis surface (Fig 5J and 5K). With the exception of *A*. *mantiquyrana*, the cuticle of the analyzed species is ornamented and may present longitudinal striations, reticles (Fig 5H), transverse striations (Fig 5E), concentric striations (Fig 5F) and irregular striations (Fig 5G).

### Column

The clinandrium is denticulate (Fig 5L) in all species of *A*. section *Pleurobotryae*, except *A*. *crepiniana*. Other species present a denticulate clinandrium, except *P*. *nemorosa*, *A*. *octophrys* and *A*. *hystrix*, where the clinandrium is smooth (Fig 5M). In frontal view the column presents epidermal cells with straight periclinal walls, except in *A*. *atropurpurea*, *A*. *hatschbachii* and *A*. *pubescens*, *A*. *ochreata*, *A*. *saurocephala* (Lodd.) Pridgeon & Chase and *A*. *teres*, where the epidermal cells are papillose. The entire surface of the column, the cuticle is ornamented with longitudinal striations in all analyzed species, except in *A*. *mantiquyrana* and *A*. *hatschbachii* which have regions smooth in the cuticle.

### Cladistic analyses

**Fig 6:**
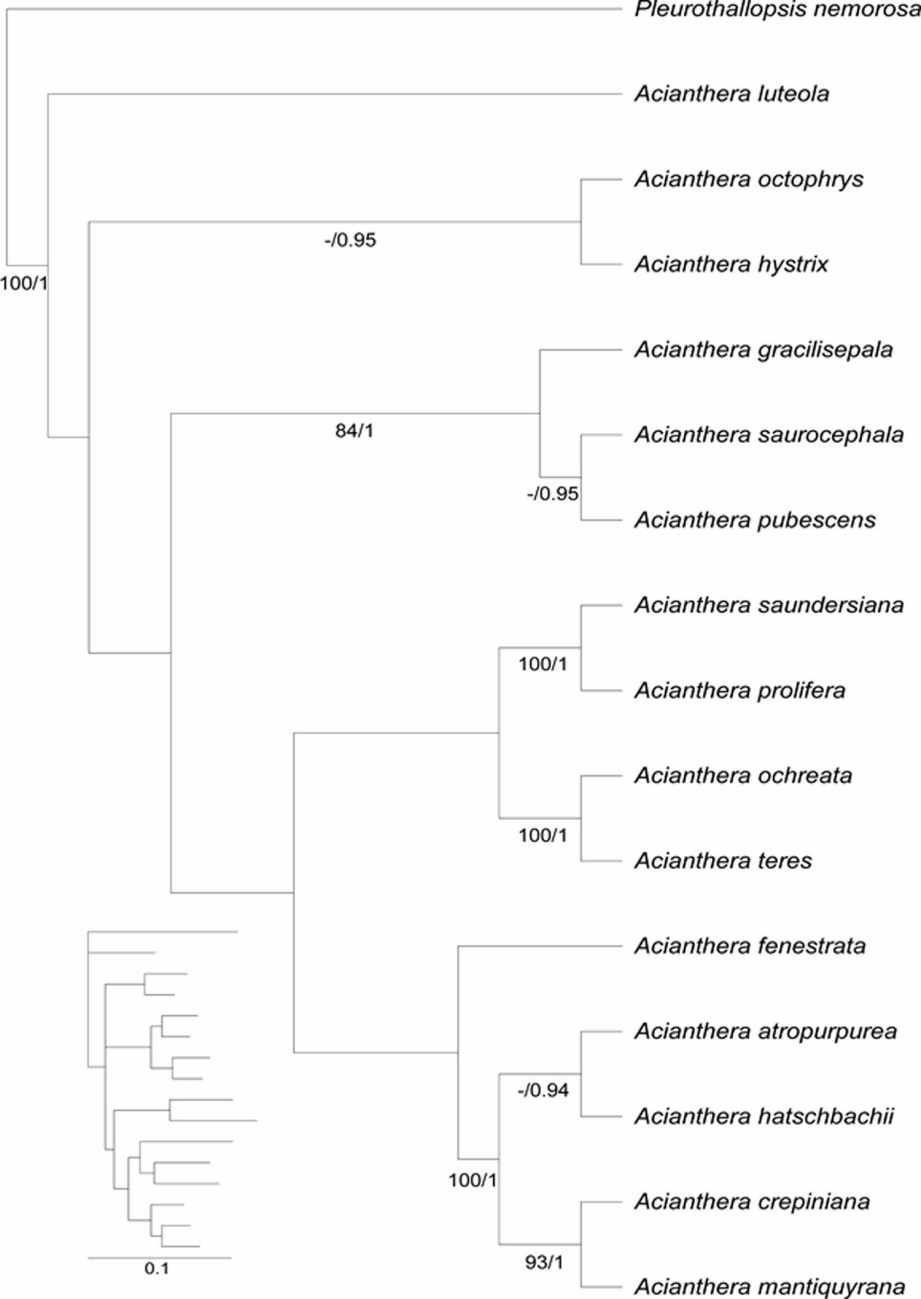
Strict consensus tree from maximum parsimony analysis. Analysis based on molecular data from cpmatK and nrITS, anatomical data for vegetative organs and floral micromorphology data. Bootstrap support and posterior probability values above 50 are indicated on the branches. Inset: majority consensus tree from Bayesian inference analysis showing proportional branch lengths.

A total of 68 anatomical characters were identified, 40 of them binary and 28 multistate, included in the morphological data matrix (S1 and S2 Appendices), and 1512 molecular characters. The matrices with vegetative anatomical characters, floral characters and molecular characters were analyzed both separately and combined. The analysis of the total evidence matrix (anatomical and molecular characters) produced one most parsimonious tree with 584 steps, consistency index (CI) 0.64, retention index (RI) 0.48 and homoplasy index (HI) 0.35 (Fig 6). Out of the 1580 characters, 1273 were constant and 148 (11%) were parsimony-informative.

**Fig 7:**
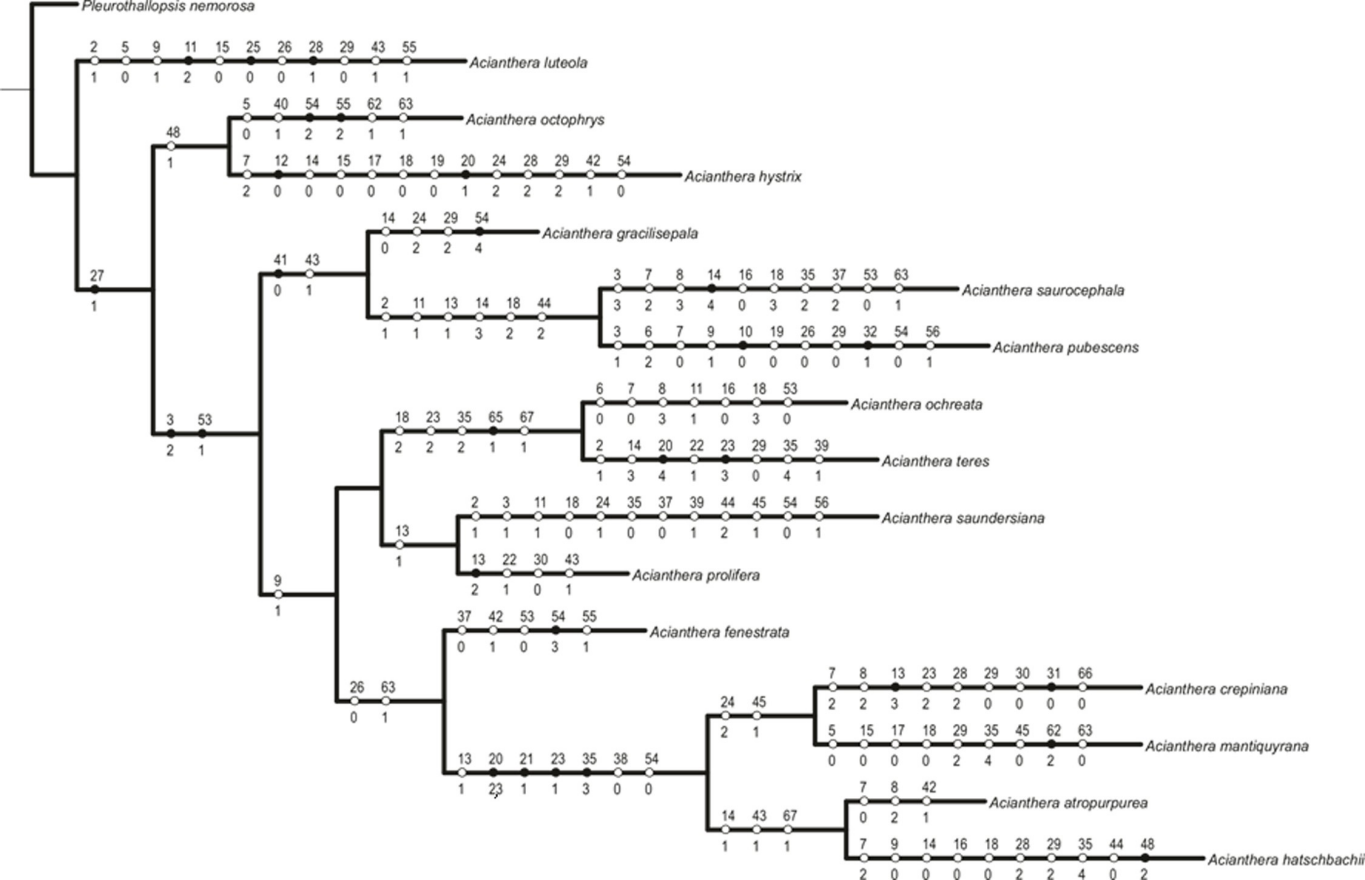
Strict consensus tree of maximum parsimony analysis as displayed in Winclada. Tree showing apomorphies (full circles) and homoplasies (open circles) for each clade with ACCTRAN optimization. Characters and character states are described in S1 Appendix.

All phylogenetic analyses recovered *Acianthera* section *Pleurobotryae* as a monophyletic group (Fig 6) with maximum support (BP = 100 / PP = 1.00). The cladistics analyses of anatomical and micromorphological data indicated 20 apomorphies from vegetative organs, being 13 autapomorphic and seven synapomorphic (Character (C) = 27, character state (S) = 1; C = 3, S = 2; C = 41, S = 0; C = 20, S = 2 and 3; C = 21, S = 1; C = 23, S = 1; and C = 35, S = 3). Four of these synapomorphies were characteristic for *A*. section *Pleurobotryae*. Among the floral characters, six autapomorphies and two synapomorphies (C = 65, S = 1; C = 53, S = 1) were identified (Fig 7).

*Acianthera* section *Pleurobotryae* presented the following synapomorphies: leaves elliptical (C = 20, S = 2) or round (C = 20, S = 3), presence of unifacial leaves (C = 21, S = 1), vascular bundles in leaves organized in concentric circles (C = 23, S = 1) and mesophyll with 28 to 30 cell layers (C = 35, S = 3). The presence of an external cortex with three layers of sclerified cells (C = 13, S = 1), absence of tracheoidal idioblasts in mesophyll (C = 38, S = 0) and labellum with round apex (C = 54, S = 0) are also a set of shared homoplastic characters for *A*. section *Pleurobotryae* (Fig 7).

The four species placed in *A*. section *Pleurobotryae* form two clades (Fig 6). In the Bayesian inference tree, the clade (*A*. *atropurpurea* + *A*. *hatschbachii*) appears as strongly supported (PP = 0.94), whereas in the parsimony analysis this clade presented low bootstrap support, sharing the following homoplasies: cortex with 8 or 9 cell layers (C = 14, S = 1), dorsal sepal margin entire (C = 43, S = 1) and papillose epidermal cells in column (C = 67, S = 1, Fig 7). The clade (*A*. *crepiniana* + *A*. *mantiquyrana*) was strongly supported in both analyses (BP = 93 / PP = 1.00), sharing the presence of leaves with papillose epidermis (C = 24, S = 2) and lateral sepals coalescent for more than 2/3 length (C = 45, S = 1) (Fig 7).

Within *Acianthera*, the species *Acianthera luteola* is sister to the remainder of the clade, and the trichome scars present in the epidermis (C = 27, S = 1) is the probable synapomorphy for the clade composed of all *Acianthera* excluding *A*. *luteola* (Fig 6).

*Acianthera gracilisepala* was recovered as sister group to the clade (*A*. *saurocephala* + *A*. *pubescens*) with moderate bootstrap support, but high posterior probability (BP = 84 / PP = 1.00) (Fig 6). This clade is characterized by the absence of idioblasts with raphides in the mesophyll (morphological apomorphy, C = 41, S = 0) and presence of a dorsal sepal with delimited margin (morphological homoplasy, C = 43, S = 1; Fig 7).

The clade (*A*. *saundersiana* + *A*. *prolifera*) is strongly supported in both analyses (BP = 100 / PP = 1.00) and the presence of three sclerified cortical cell layers in the ramicaul (C = 13, S = 1) is a shared morphological homoplasy (Fig 7). *Acianthera teres* and *A*. *ochreata* constitute a clade sister to (*A*. *saundersiana* + *A*. *prolifera*) also with strong support values (BP = 100 / PP = 1.00), characterized by the presence of 26 to 28 vascular bundles in the ramicaul (C = 18, S = 2), vascular bundles in leaves organized in one row (C = 23, S = 0), mesophyll with 25 or 26 cell layers (C = 35, S = 2) and column with papillose cells (C = 67, S = 1) (Fig 7). The presence of epidermal papillose cells in the petals is synapomorphic for the clade (C = 65, S = 1, Fig 7).

## Discussion

The analysis of molecular data combined with anatomical and micromorphological data of *Acianthera* section *Pleurobotryae* allowed us to identify shared anatomical characters observed exclusively in these species, besides supporting the internal relationships suggested by the molecular data set [15]: unifacial leaves, round or elliptical in cross-section, round leaves with vascular bundles organized in concentric circles, and mesophyll with 28 to 30 cell layers. Cladistic analyses also revealed shared homoplastic characters, such as the presence of three sclerified cortical cell layers in the ramicaul, absence of tracheoidal idioblasts in the mesophyll and labellum with round apex.

All analyzed species, epiphytic or rupicolous, have thin roots with bi-stratified velamen, indicating that this is a characteristic of the genus, not influenced by habitat variations. The presence of bi-stratified velamen was previously recorded for *A*. *teres* [26], A. *pubescens* [27] and *A*. *prolifera* [28]. In Pleurothallidinae, the roots may have one, two or many layers of velamen [6]; the species representative of the outgroup, *Pleurothallopsis nemorosa*, also presented bi-stratified velamen.

The species in *Acianthera* section *Pleurobotryae* present an elongated and thin ramicaul with sclerified cortex layers. The presence of three cell layers is one of the homoplasies of the clade observed in the two species of the section that present the largest, pending leaves (*A*. *atropurpurea* and *A*. *hatschbachii*) and in *A*. *mantiquyrana*, which presents smaller, erect leaves. The presence of five sclerified layers in the ramicaul is an autopomorphy of *A*. *crepiniana*, which presents large, erect leaves. Sclerenchyma confers resistance to plant organs [58], and the abundant sclerenchyma found in Pleurothallidinae is important to support the ramicaul [59]; thus, the higher number of sclerified cell layers in *A*. *crepiniana* may be associated with the need for mechanical support. *Acianthera hystrix* did not present sclerified cell layers in the ramicaul; the ramicaul in this species is reduced, with reptant growth and overlayed leaves, which decreases the need for sclerified support tissues.

The leaves in *Acianthera* section *Pleurobotryae* are cylindrical or laterally compressed [14] and round or elliptical in cross-section. The presence of round leaves in *A*. *mantiquyrana*, *A*. *hatschbachii* and *A*. *atropurpurea* is synapomorphic for these species and the presence of elliptical leaves in *A*. *crepiniana* is autapomorphic. Ancestral state reconstruction analyses indicated that elliptical (laterally compressed) leaves of *A*. *crepiniana* evolved from round (cylindrical) leaves observed in the other species of this section. This evolutionary pattern of leaf blades has also been observed in *Juncus*, where laterally compressed leaves evolved from cylindrical leaves through a flattening process of the leaf blade that is genetically regulated [60, 61]. Since the species of *A*. section *Pleurobotryae* are epiphytic and occur in the Atlantic Forest [14], where the capture of sunlight can be difficult, the change from cylindrical to laterally compressed shape could be an adaptation for an increased area of exposure of the leaf, thus improving the efficiency of capture of sunlight [61, 62].

The organization of vascular bundles in concentric circles is a synapomorphy in the species of *A*. section *Pleurobotryae* that present cylindrical leaves. In *A*. *crepiniana*, the vascular bundles are disposed in two parallel rows. The arrangement of vascular bundles in circles or opposed pairs, where the xylem is disposed towards the middle of the lateral blade and the phloem, outwards, is characteristic for unifacial leaves [63, 64]. The round-sulcate leaves of *A*. *teres* are bifacial or dorsiventral, the abaxial surface surrounding the leaf almost completely and the adaxial surface being restricted to the sulcus. Among the species analyzed, only species of *A*. section *Pleurobotryae* presented unifacial leaves, which is considered a synapomorphy for this group. Unifacial leaves appeared several times in the evolutionary history of the monocotyledons and are characterized by the abaxialization of the leaf blade and consequent suppression of the adaxial surface [61, 63]. In Epidendroideae (Orchidaceae), unifacial leaves were observed in epiphytic species of Dendrobieae and, together with other anatomical foliar characters, this feature supported the monophyly of *Dendrobium* section *Rhizobium* [65]. In Malaxideae (Epidendroideae), molecular analyses showed that unifacial leaves appear in all epiphytic species, forming a clade [66].

Tracheoidal elements or tracheoidal idioblasts [67] are distinct from others in shape, size, content, and wall structure, and are common in Orchidaceae [5, 26, 68]. It has been hypothesized that the function of these structures is associated with the mechanical support of the surrounding parenchymal tissues, since the wall with secondary thickening can prevent collapse during desiccation [68]. Tracheoidal idioblasts with helical thickening were recorded in several epiphytic and terrestrial species, and appeared several times in the evolutionary history of Orchidaceae [68]. These structures are present in the mesophyll of the flat, semi-flat and round-sulcate leaves of the other species analyzed. The absence of tracheoidal idioblasts with helicoidal thickening in the mesophyll is a shared homoplasy for *A*. section *Pleurobotryae*, which were the only species in this genus where circular or elliptical leaves were observed. Thus, the leaf shape of these species may provide greater mechanical resistance to desiccation, and may have favored the loss of these structures.

The main chemical constituent of the wall thickening of tracheoidal idioblasts is still cause for discrepancies in the literature. Pridgeon [6] did not record the presence of lignin and stated that the cell wall of these thickenings is exclusively cellulosic, while Olantuji & Neguin [68] and Scatena & Nunes [26] argued that the wall thickenings of these cells did present lignin in its constitution. We recorded autofluorescence in the wall thickenings of the idioblasts, demonstrating the deposition of lignin on the helical walls of these structures through analysis with fluorescence microscopy [39]. Thus, we have verified that lignin is the main component of the wall thickenings present in the tracheoidal idioblasts of the leaves of *Acianthera* analyzed in this study.

The presence of a papillose leaf epidermis in *A*. section *Pleurobotryae* is a homoplasy shared between species of the clade formed by *A*. *crepiniana* and *A*. *mantiquyrana*. In Pleurothallidinae, the papillose epidermis has also been observed in *Dresslerella* [23]. These structures may play important roles in the uptake or dissipation of water on the surface of organs [43].

Among the flower morphological and micromorphological characters, eight (34.7%) were apomorphies (Fig 7) mainly of labellum features. Epistomatic sepals are autapomorphic for *A*. *hatschbachii*; other species may present stomatal pores but their position is variable. In the cladistic analyses, the presence of hypostomatic sepals is a synapomorphy for the clade formed by the representative of *Acianthera* section *Tomentosae* and *Acianthera* section *Crinitae*. The presence of stomatal pores in sepals is associated with emissions of volatile compounds by osmophores [69], floral tissues that are specialized for the synthesis and secretion of fragrances [70]. *Acianthera* and other Pleurothallidinae species are pollinated by Diptera [3], and osmophores are important for the ecology of pollination as they have a role on the attraction of specific pollinators [71].

The reduced petals are a morphological feature characteristic of *Acianthera* [8]. In the analyzed species, the linear petals of *A*. *mantiquyrana* are autapomorphic, while the papillose regions observed in petals of *A*. *ochreata* and *A*. *teres* are synapomorphic for these species, indicating petals may present morphological and micromorphological characters useful for the delimitation of species and sections within the genus.

Labellum apex shape appeared in the phylogenetic analyses as autapomorphic for *Acianthera octophrys* (C = 54, S = 2), *A*. *gracilisepala* (C = 54, S = 4) and *A*. *fenestrata* (C = 54, S = 3). *Acianthera octophrys* presents an unguiculate labellum, with retuse apex (C = 54, S = 2), while the species of *Acianthera* section *Pleurobotryae* present an unguiculate labellum with round apex (C = 54, S = 0). This supports the removal of *A*. *octophrys* from section *Pleurobotryae* [15]. Shared morphological features of the labellum may be convergent in response to selective pressure from similar pollinators [45].

The presence of papillose cells in the column is one of the homoplastic floral characters shared by the clade (*A*. *atropurpurea* + *A*. *hatschbachii*). Petals and columns with papillose regions are also present in the (*A*. *ochreata* + *A*. *teres*) clade. In flowers of other myophilic species, *Bulbophyllum* Thouars, papillose regions presented lipids and were involved in the process of volatile emission [21]. In Pleurothallidinae, papillose regions of the labellum are known to emit volatiles and act as osmophores [72]; however, there are few records for such regions in petals and columns in *Acianthera*. Flower papillae may present various shapes in Orchidaceae, as observed in the species studied here, and have various functions in the pollination process: attracting pollinators by the secretion of viscous materials and waxes [73], guiding pollinators by tactile stimuli, or protecting the floral parts, preventing desiccation [44].

In the cladistic analyses performed here, the representative species of *A*. section *Cryptophoranthae* appears as sister group of *A*. section *Pleurobotryae*, even though it presents a number of morphological differences in both vegetative and reproductive organs. *Acianthera fenestrata* shares with species of *A*. section *Pleurobotryae* the presence of stomata with five subsidiary cells and petals with entire margins, the positioning of this species in molecular analyses is different [15], reinforcing the importance of anatomical and micromorphological studies for the taxonomy and systematic of the genus.

*Acianthera* section *Sulcatae* appears as sister to all other sections of the genus. In molecular analyses, the specie representative of this section appears as sister of the *A*. section *Pleurobotryae* with low support [15]. *Acianthera luteola* did not present scars of trichomes in the leaves, unlike the other species analyzed. In the early stages of development, the leaves may present with colleters, trichomes that initiate the secretion phase before leaf expansion, helping growth and preventing desiccation [74]. This is the first record of trichome scars on Pleurothallidinae leaves. Further anatomical studies of leaves at different stages of development could reveal new information about morphology and function of these trichomes.

In the molecular analyses [15] and in the combined analyses performed here, the representatives species of the *Acianthera* section *Tomentosae* and *A*. section *Crinitae* appears in a clade with high support, with the presence of hypostomatic sepals. The rupicolous species of *Acianthera* section *Tricarinatae* appeared as sister group of the epiphytic species of *Acianthera* section *Sicariae*, but the analyses did not indicate any shared vegetative, anatomical and floral micromorphology characters among these groups. Studies with a representative number of species could clarify the phylogenetics relations between these sections.

*Acianthera gracilisepala* appeared as sister to the clade *Acianthera* section *Acianthera*. The placement of *A*. *gracilisepala* in section *Acianthera* was suggested by Rodrigues et al. [15] through molecular analyses and confirmed in the present study, based on anatomical and micromorphological characteristics such as absence of idioblasts with raphides on leaves and presence and dorsal sepal with delimited margins.

Several species originally described as *Pleurothallis* are currently placed in *Acianthera*, and in such cases, characters from vegetative organs may be more phylogenetically informative than floral characters [75]. Floral features may not be reliable to establish phylogenetic relationships, since similarities between flowers of *Pleurothallis* (*Acianthera johannensis*, *A*. *teres* e *A*. *ochreata*) may be due to convergence, as these species may share the same pollinators [76]. In this study, the homoplasy index for characters from vegetative organs was 0.4 and for floral organs, 0.6, supporting the argument that characters based on vegetative anatomy are more conserved than the floral micromorphology characters for the species studied, and may be more useful to infer relationships within *Acianthera*. Thus, molecular, anatomical and micromorphological studies of more species of *Acianthera*, with a more thorough sampling of other sections, could reveal more diagnostic anatomical, morphological and micromorphological characters for each section and help in the interpretation of the evolutionary history of these species.

## Conclusion

*A*. section *Pleurobotryae* is a strongly supported monophyletic group with the following synapomorphies: leaves unifacial, round or elliptical in cross-section, round leaves with vascular bundles organized in concentric circles, and mesophyll with 28 to 30 cell layers. All synapomorphies and most apomorphies for *A*. section *Pleurobotryae* were found in vegetative organs. This supports the hypothesis that vegetative organs are more stable evolutionarily compared to flowers, maybe due to the diversification of pollinator attraction mechanisms. Within the section, the species that were most morphologically distinct were *A*. *crepiniana* and *A*. *mantiquyrana*, although this clade presented higher support in molecular analyses. We consider that the elliptical leaves in *A*. *crepiniana* derived from round leaves observed in the other representatives of this section, as an adaptation to increase the area of exposure of the leaf and better the efficiency of capture of sunlight. We found that the presence of papillose regions in both vegetative and floral organs indicated that micromorphological characters are also useful for the delimitation of species and sections within the genus.

## Supporting information

S1 Appendix List of anatomical and micromorphological characters. (DOCX)

S2 Appendix Cladistic analysis characters matrix. (DOCX)
