# Supplementary material for: Evolution of anatomical characters in Acianthera section Pleurobotryae (Orchidaceae: Pleurothallidinae)
Source: PLoS One. 2019 Mar 13;14(3):e0212677. doi: 10.1371/journal.pone.0212677 (PMC6415883; doi:10.1371/journal.pone.0212677)
Supplement: S1 Appendix — (DOCX) [file pone.0212677.s001.docx]

**S1 Appendix.** List of anatomical and micromorphological characters.

CHARACTERS AND CHARACTER STATES

1. Root, bi-stratified velamen: (0) absent, (1) present.

2. Root, exodermis, cell wall thickening: (0) inverted-U shaped, (1) O-shaped.

3. Root, cortex, number of layers: (0) up to 6, (1) 7, (2) 8, (3) 9, (4) 10.

4. Root, cortex, presence of reticulate idioblasts: (0) present, (1) absent.

5. Root, endodermis, type of wall thickening: (0) U-shaped, (1) O-shaped.

6. Root, endodermis, wall thickness: (0) fine to thick, (1) very thin, (2) very thick.

7. Root, endodermis, number of passage cells: (0) 1, (1) 2, (2) 3.

8. Root, number of protoxylem poles: (0) 5, (1) 7 to 9, (2) 10, (3) 15 to 16.

9. Root, presence of raphides: (0) absent, (1) present.

10. Root, medulla, type of wall: (0) primary, (1) secondary.

11. Ramicaul, shape in cross section: (0) round, (1) round sulcate, (2) triangular.

12. Ramicaul, presence of external sclerified cortex: (0) absent, (1) present.

13. Ramicaul, number of sclerified cortical layers: (0) up to 2, (1) 3, (2) 4, (3) 5.

14. Ramicaul, total number of cell layers in cortex: (0) up to 7, (1) 8 or 9, (2) 10 or 11, (3) 13, (4) 16.

15. Ramicaul, presence of aerenchyma: (0) absent, (1) present.

16. Ramicaul, presence of starch: (0) absent, (1) present.

17. Ramicaul, presence of sclerifiedinterfascicularfundamental tissue: (0) absent, (1) present.

18. Ramicaul, number of vascular bundles: (0) up to 15, (1) 18 to 25, (2) 26 to 28, (3) 40 to 50.

19. Ramicaul, pith, type of cell walls: (0) primary, (1) secondary.

20. Leaf, leaf blade shape in cross section: (0) semi-flat, (1) flat, (2) elliptical, (3) round.

21. Leaf, configuration: (0) bifacial, (1) unifacial.

22. Leaf, stomata distribution: (0) hypostomatic, (1) amphistomatic.

23. Leaf, vascular bundles organization: (0) 1 line parallel to larger axis of the leaf, (1) concentric circle, (2) 2 lines parallel to the larger axis of the leaf, (3) 3 lines parallel to the larger axis of the leaf.

24. Leaf, epidermal surface in frontal view: (0) smooth, (1) verrucous, (2) papillose.

25. Leaf, shape of epidermal cell wall in frontal view: (0) curved, (1) straight.

26. Leaf, number of subsidiary cells in stomatal complex: (0) up to 5, (1) 6.

27. Leaf, presence of trichome scars: (0) absent, (1) present.

28. Leaf, type of epicuticular wax: (0) smooth layer, (1) granules, (2) crust.

29. Leaf, shape of epidermal cells in cross section: (0) dome-shaped, (1) polygonal, (2) papillose.

30. Leaf, presence of adaxial hypodermis: (0) absent, (1) present.

31. Leaf, presence of abaxial hypodermis: (0) absent, (1) present.

32. Leaf, abaxial hypodermis, number of layers: (0) 1layer, (1) 2 layers.

33. Leaf, abaxial hypodermis, cells with thickened walls: (0) absent, (1) present.

34. Leaf, presence of heterogeneous chlorophyll parenchyma: (0) absent, (1) present.

35. Leaf, number of layers in mesophyll: (0) up to 15, (1) 17 to 20, (2) 25 to 26, (3) de 28 to 30, (4) 35 or 36.

36. Leaf, distinctly modified epidermal and mesophyllcells in midrib: (0) absent, (1) present.

37. Leaf, aquiferous parenchyma cell organization: (0) one row, adaxial, (1) two rows separated by a row of chlorophyll parenchyma, adaxial, (2) throughoutmesophyll.

38. Leaf, mesophyll, presence of tracheoidalidioblasts with helicoidal thickening: (0) absent, (1) present.

39. Leaf, shape of tracheoidalidioblasts with helicoidal thickening: (0) cylindrical, (1) globose.

40. Leaf, presence of idioblasts with prismatic crystals: (0) absent, (1) present.

41. Leaf, presence of idioblasts with raphides: (0) absent, (1) present.

42. Dorsal and lateral sepals, coalescence: (0) free, (1) partially coalescent.

43. Dorsal sepals, adaxial surface, type of margin: (0) entire, (1) delimited.

44. Dorsal sepals, adaxial surface, margin ornamentation: (0) glabrous, (1) ciliate, (2) papillose.

45. Lateral sepals, degree of coalescence: (0) up to 1/3 length, (1) over 2/3 length, (2) completely coalescent.

46. Lateral sepals, presence of mentum: (0) absent, (1) present.

47. Sepals, shape of epidermal cells: (0) polygonal, (1) elongate polygonal.

48. Sepals, stomata distribution: (0) amphistomatic, (1) hypostomatic, (2) epistomatic.

49. Sepals, apex cells differentiated: (0) absent, (1) present.

50. Sepals, cell types: (0) straight wall cells, (1) papillae, (2) trichomes.

51. Sepals, papillae types: (0) simple, (1) obpyriform, (2) conical, (3) ovate, (4) obovate, (5) with aciculate apex.

52. Trilobed labellum: (0) absent, (1) present.

53. Labellum, shape of lateral lobes: (0) round, (1) apiculate.

54. Labellum, apex shape: (0) round, (1) obtuse, (2) retuse, (3) truncate, (4) acute.

55. Labellum, margin: (0) serrate, (1) entire, (2) ciliate.

56. Labellum, adaxial surface, homogeneous surface: (0) absent, (1) present.

57. Labellum, epidermal cells shape: (0) elongate polygonal, (1) irregular, (2) polygonal.

58. Labellum, cell types: (0) straight wall cells, (1) papillae, (2) trichomes.

59. Labellum, papillae types: (0) simple, (1) conical, (2) ovate, (3) imbricate, (4) obpyriform.

60. Labellum, cuticle type: (0) smooth, (1) ornamented.

61. Labellum, cuticle ornamentation: (0) longitudinal striations, (1) irregular striations, (2) concentric striations, (3) transverse striations, (4) reticulate.

62. Petal, shape: (0) oblanceolate, (1) deltoid, (2) linear.

63.Petal, margin: (0) serrate, (1) entire.

64. Petal, shape of epidermal cells: (0) elongate polygonal, (1) polygonal.

65. Petal, type of epidermal periclinal wall: (0) straight wall cells, (1) papillae.

66. Column, margin type: (0) smooth, (1) denticulate.

67. Column, type of epidermal periclinal wall: (0) straight wall cells, (1) papillae.

68. Column, cuticle type: (0) smooth, (1) ornamented.
