# Supplementary material for: Evolution of anatomical characters in Acianthera section Pleurobotryae (Orchidaceae: Pleurothallidinae)
Source: PLoS One. 2019 Mar 13;14(3):e0212677. doi: 10.1371/journal.pone.0212677 (PMC6415883; doi:10.1371/journal.pone.0212677)
Supplement: S2 Appendix — (DOCX) [file pone.0212677.s002.docx]

**S2 Appendix:** Cladistic analysis characters matrix.

**S2 Appendix:** Characters matrix, continued.
